# Phytochemical profiles of honey bees (*Apis mellifera*) and their larvae differ from the composition of their pollen diet

**DOI:** 10.1098/rsos.231654

**Published:** 2024-09-25

**Authors:** Nanna Hjort Vidkjær, Bente B. Laursen, Per Kryger

**Affiliations:** ^1^ Department of Biology, University of Copenhagen, Copenhagen, Denmark; ^2^ Department of Agroecology, Aarhus University, Slagelse, Denmark

**Keywords:** bee health, flavonoids, alkaloids, phenolic acids, post-ingestion metabolism, plant secondary metabolites

## Abstract

Pollen and nectar consumed by honey bees contain plant secondary metabolites (PSMs) with vital roles in plant–insect interactions. While PSMs can be toxic to bees, they can also be health-promoting, e.g. by improving pesticide and pathogen tolerances. As xenobiotics, PSMs undergo post-ingestion chemical modifications that can affect their bioactivity and transmission to the brood. Despite the importance of understanding honey bee PSM metabolism and distribution for elucidating bioactivity mechanisms, these aspects remain largely unexplored. In this study, we used HPLC-MS/MS to profile 47 pollen PSMs in honey bees and larvae. Both adult bees and larvae had distinct PSM profiles that differed from their diet. This is likely due to post-ingestion metabolism and compound-dependent variations in PSM transmission to the brood via nurse bee jelly. Phenolic acids and flavonoid aglycones were most abundant in bees and larvae, whereas alkaloids, cyanogenic glycosides and diterpenoids had the lowest abundance despite being consumed in higher concentrations. This study documents larval exposure to a variety of PSMs for the first time, with concentrations increasing from early to late larval instars. Our findings provide novel insights into the post-ingestion fate of PSMs in honey bees, providing a foundation for further exploration of biotransformation pathways and PSM effects on honey bee health.

## 1. Introduction

Plants produce an extraordinary array of secondary metabolites (PSMs, also known as plant specialized metabolites) with important roles in defences against herbivores and pathogens, as well as in plant signalling [1,2]. These phytochemicals are also present in the pollen and nectar consumed by bees [3,4], but their role in plant–pollinator interactions is less obvious. PSMs exhibit a wide range of bioactivities, including antiviral and antimicrobial properties. While some PSMs can be toxic [5,6], co-evolutionary mutualism seems to enable insect pollinators to derive benefits from these compounds, particularly in their defences against pathogens [7–10]. Recent pollinator declines have led to an increasing number of studies on how PSMs influence bee health. Although only a few PSMs have been tested, these studies suggest a multifaceted role including toxic, antiviral [11,12] and immunostimulatory activity [13–15], improved pathogen defences [16–21], increased longevity [17,18] and changes in gut microbial composition and abundance [22]. Some flavonoids and phenolic acids enhance honey bees’ pesticide tolerance and detoxification by inducing the expression of genes regulating the cytochrome P450 detoxification system [10,15,23–26]. PSMs have primarily been tested on adult bees, leaving health effects and toxicity levels in the brood underexplored. For species like honey bees, which feed their larvae glandular secretions rather than pollen and nectar directly, brood PSM exposure remains largely unexplored as it depends on the extent to which pollen and nectar PSMs are transferred to these secretions.

PSMs are xenobiotics to bees and metabolized after ingestion by the bees’ own enzymatic detoxification systems [27,28] with contributions from gut microorganisms [29,30]. Despite the increasing amounts of research showing diverse PSM functions in bee health, post-ingestion chemical reactions are poorly understood in bees as well as in other insects. Metabolization pathways, detailing the transformation steps of PSMs, likely differ based on molecular structure and properties similar to mammalian PSM metabolism [31] but are known for only a few PSMs in a small number of species. Knowing how various PSMs are taken up, metabolized and distributed within bees is important for understanding and predicting PSM bioactivities and unravelling mechanisms underlying specific health effects. This was for instance recently exemplified in bumble bees for a PSM with antiparasitic activity where the active compound was a metabolite formed in the bees while the ingested parent PSM was inactive [32]. This also emphasizes that laboratory assays testing the direct activity of PSMs against cultured pathogens (e.g. [32]) may not accurately reflect the *in vivo* effects, as these assays do not account for post-ingestion metabolization or gut PSM uptake and distribution within bees.

For honey bees, which feed their larvae jelly produced in the specialized hypopharygeal glands of nurse bees [33,34], the post-ingestion fate of PSMs can also influence transmission to the brood. Early larval instars receive only jelly, while older larvae destined to become worker bees also receive small amounts of pollen and honey [33,34]. Nurse bees must consume large quantities of bee bread [33]—a mix of pollen, honey, nectar and glandular secretions [35]—to produce jelly. This creates a potential, but underexplored, pathway for PSM transmission to the brood via jelly. The exact mechanism of PSM transfer from diet to jelly remains unknown but the PSM profile of jelly likely differs from that of the diet. Some PSMs may undergo metabolization before being passed to the glands, while others may not be transmitted at all. To date, only a few studies document PSMs in jelly [36,37] while none have tested the PSM content in honey bee brood. If the brood is exposed to a wide range of PSMs, understanding how PSMs influence larval health is essential—not only for assessing impacts on the individual larva but also for understanding the effects on colony health and rejuvenation.

In this study, we exposed miniature honey bee colonies to a field-realistic clover pollen diet containing flavonoids and phenolic acids, which are ubiquitous in pollen and nectar. To introduce a broader range of dietary PSM compound classes, we fortified this diet with alkaloids, diterpenoids and cyanogenic glycosides produced by fewer plant species. Our objectives were to (i) identify dietary PSMs present in adult honey bees and larvae, (ii) determine how the PSM composition changes across larval developmental stages, and (iii) compare PSM profiles between adult bees and larvae. We hypothesized that the PSM profiles of both adult bees and larvae would differ qualitatively from the diet and vary across different PSM molecular structures due to metabolization differences. Our aims were to determine the PSM content of larvae for the first time and provide new insights into the transmission of PSMs from diet to adult bees and brood, including comparing the transmission of PSMs that are omnipresent in pollen and nectar with compounds produced by fewer plant species. The results provide new insights to guide detailed mapping of key PSM metabolization pathways and PSM impact on larval health.

## 2. Material and methods

### 2.1. Chemicals

Details about the chemicals used for extractions and as analytical standards for the HPLC-MS/MS analyses are included in the electronic supplementary material (table S1).

### 2.2. Honey bees

Six small *Apis mellifera* L. colonies (~750–1100 bees per colony) were kept in miniature hives (24 × 15 × 15 cm) (Mini-mating nucs; Apidea, Switzerland) consisting of a queen, worker bees and brood [38,39]. The hives originated from sister queens hatched in June and mated in early July, establishing small colonies for this experiment in September. The six colonies were selected based on size similarity and comparable amounts of brood. Three hives were used for the feeding experiment focusing on PSMs in larval stages (§2.4), while the other three provided newly hatched bees for a separate experiment measuring dietary PSMs in adult bees (§2.5). All miniature hives were kept in the apiary of the Department of Agroecology, Aarhus University, Flakkebjerg, Denmark.

### 2.3. Pollen diets

The diet consisted of white clover (*Trifolium repens*) pollen fortified with a series of PSMs. Clover pollen PSMs have not previously been analysed, but existing knowledge on PSMs in white clover leaves, stems, flowers and roots suggests that the pollen contains a range of flavonoids and phenolic acids [40–45] (see §2.6.1). To expand the range of compound classes, we fortified the clover pollen with six additional PSMs: gelsemine (indole alkaloid), atropine (tropane alkaloid), senkirkine (pyrrolizidine alkaloid), amygdalin (cyanogenic glycoside), triptolide (diterpenoid epoxide) and methyllycaconitine (diterpenoid alkaloid) (systematic names are included in electronic supplementary material, table S2). These compounds were selected based on three criteria: (i) they represent PSM compound classes not naturally present in white clover, (ii) their natural concentrations in pollen and/or nectar are reported in the literature (see [46–53]), and (iii) they are commercially available as isolation from natural sources in the needed amounts was not feasible. Toxic effects have been reported for several of these PSMs [5,51,54–56], with details on toxicity levels and bioactivities in adult bees summarized in Vidkjær *et al*. [57]. No studies have tested these compounds in bee larvae, and larval toxicity levels, health effects and capacity for metabolizing these compounds are unknown. The concentrations in the fortified diet were within the natural range for pollen and/or nectar and below toxicity thresholds for adult honey bees (summarized in Vidkjær *et al*. [57]).

Corbicular white clover pollen was collected with traps mounted at the entrances of honey bee hives located in clover fields at the Department of Agroecology, Aarhus University, Flakkebjerg, Denmark, and kept at −20°C. Palynological analyses confirmed that the pollen was 92% *Trifolium repens*. To prepare experimental diets, 15 g of corbicular pollen was mixed with 1 ml Apiinvert^®^ (28% water and 72% sugar: 30% saccharose, 31% dextrose, 39% fructose) and 0.4 ml water using a pestle and mortar. The fortified pollen diet was then prepared by adding 1.5 mg gelsemine, 0.3 mg atropine, 0.75 mg amygdalin, 0.045 mg senkirkine, 0.015 mg methyllycaconitine and 0.015 mg triptolide. These compounds were first dissolved in either methanol, ethanol or ethyl acetate to create stock solutions (300 µg ml^−1^ to 22.5 mg ml^−1^) kept at −20°C. When the diet was prepared, the individual PSMs were mixed by adding 50–82 µl of each stock solution (463 µl in total) to 0.5 ml ethanol. This mixture was then added to the clover pollen paste and thoroughly mixed with a pestle and mortar to ensure uniform distribution. Approximately 5 g of the resulting pollen paste was then transferred to sterile plastic well plates and left at room temperature for 1 h to allow solvent evaporation. The well plates were then attached to new, unused wooden frames without wax combs with elastic bands and mounted in the miniature hives (electronic supplementary material, figure S1). The pollen diets were prepared and replaced every 24 h to minimize compound degradation and monitor food intake.

### 2.4. Feeding experiments with small honey bee hives to measure plant secondary metabolites in larval stages

Three miniature hives (see §2.2), H1, H2 and H3, with adult bees, brood and a queen, were used for this experiment. One week before the experiment started, the nectar frames were removed and replaced with plastic well-plates containing PSM-free Apifonda^®^ (85% sugar, mainly saccharose; approx. 300 g per hive). Three days before the experiment, hive entrances were closed, and pollen frames were removed to prevent unintended PSM exposure. The experiment began with feeding the sealed hives a pure clover pollen diet for 2 days. On day three, feeding with the fortified clover pollen diet started and continued for 3 days. The experiment ended on day six. By this time, the hives had been sealed for 8 days, the average duration for a honey bee egg to hatch and reach the pupae stage.

To collect the larvae, the hives were anaesthetized with CO_2_, and the number of bees counted (H1: 1120 bees; H2: 901 bees; H3: 747 bees). Larvae in the third, fourth and fifth instar (separated based on size) were gently collected, washed with Milli-Q water to remove PSM-containing jelly from the cuticle, and sacrificed by freezing. The numbers of larvae collected from each nucleus hive were H1: third instar, 85; fourth instar, 33; fifth instar, 35; H2: third instar, 51; fourth instar, 42; fifth instar, 19; H3: third instar, 37; fourth instar, 26; fifth instar, 17. Given the larvae’s age distribution, they must have hatched after the hives were sealed within the timeframe of the experiment. Five fifth instar, five fourth instar and ten third instar larvae were kept for analyses of pollen grains in their gut. For chemical analyses, three sub-samples of each larval instar were prepared for each hive by pooling the following numbers of larvae: third instar, *n* = 9; fourth instar, *n* = 7; and fifth instar, *n* = 4. This resulted in nine larval samples per hive and a total of 27 samples for chemical analyses. These samples were freeze-dried for 72 h (Heto Drywinner, Birkerød, Denmark). Metal beads were then added to the dried larval samples, which were pulverized by vibration for 30 s at 1500 r.p.m. using a Geno/Grinder (SPEX Sample Prep 2010, Metuchen, NJ, USA).

### 2.5. Feeding experiment with adult honey bees

PSM profiles of adult honey bees were assessed in a separate setup. Flavonoids and phenolic acids are omnipresent in pollen and nectar [26,58], and our previous analyses (unpublished data) show that adult honey bees retain these compounds even after consuming a pure PSM-free sucrose diet for several days. In the experimental miniature hives (§2.4), we could not distinguish young adult bees hatched after the hives were sealed from older foragers pre-exposed to pollen and nectar that were in the hives before the beginning of the experiment. To minimize background levels of flavonoids and phenolic acids and to analyse PSMs in young bees that have the highest pollen consumption during their maturation into nurses, we performed this experiment in a separate setup.

Newly hatched adult bees for this experiment came from the three miniature colonies H4, H5 and H6 (see §2.2). Before starting the feeding experiment, we sealed the hives and replaced pollen and nectar frames with PSM-free Apifonda^®^ (85% sugar, mainly saccharose; approx. 300 g per hive) and pure clover pollen diet (see §2.3). After two weeks, frames with capped brood cells were moved to a dark incubator at 35°C and 30–50% humidity. Newly emerged bees were collected after 2 days and combined into one queenless miniature hive (24 × 15 × 15 cm) (Mini-mating nuc; Apidea, Switzerland) containing bees from H4, H5 and H6. A queen pheromone strip was used to simulate the presence of a queen. To replicate PSM exposure from the larval experiment (§2.4), the young adult bees were fed pure clover pollen diet for 2 days, followed by fortified clover pollen diet for 3 days, with additional access to Apifonda^®^. Diets were replaced daily to prevent PSM degradation and measure pollen intake. The mean daily intake was 5.6 ± 2.9 mg per bee. On day six, feeding was terminated, and the bees were anaesthetized with CO_2_, counted (285 bees) and sacrificed by freezing. Bees were washed with Milli-Q water to remove pollen diet residues from the cuticle, then divided into five sub-samples of 50 bees each. These samples were freeze-dried for 72 h (Heto Drywinner, Birkerød, Denmark), then metal beads were added, and the samples were pulverized by vibration for 30 s at 1500 r.p.m. using a Geno/Grinder (SPEX Sample Prep 2010, Metuchen, NJ, USA).

### 2.6. Sample extraction

#### 2.6.1. Pollen

The PSM composition of white clover pollen has not previously been assessed. The 29 flavonoids and 12 phenolic acids targeted by our HPLC-MS/MS methods (see electronic supplementary material, table S2, for a complete list including systematic names) were selected based on existing knowledge of PSMs in white clover plants [42,45,59].

Flavonoids and phenolic acids were extracted from 100 mg clover pollen with 4 ml of 7:3 methanol:water by ultrasonication for 1 h at 30°C. The extract was centrifuged (12 min, 4°C, 4500 r.p.m.), the supernatant was removed and the extraction was repeated. The two extracts were combined and stored at −20°C. The extraction protocol was based on quantitative analyses of flavonoids and phenolic acids in pollen [60] and bee bread [61] established by others.

The six additional PSMs artificially added to the pollen diet were not quantified as they were added to the diet in specific known concentrations. These concentrations were gelsemine (100 ng mg^–1^), atropine (20 ng mg^–1^), amygdaline (50 ng mg^–1^), senkirkine (3 ng mg^–1^), methyllycaconitine (1 ng mg^–1^) and triptolide (1 ng mg^–1^).

#### 2.6.2. Adult honey bees

Extractions of gelsemine, atropine, amygdaline, senkirkine, methyllycaconitine and triptolide from the five pools of adult honey bees followed previously published and validated protocols using 35 mg of the dried bee samples [57].

Flavonoids and phenolic acids were extracted from 35 mg dried bee samples with 1 ml of 7:3 methanol:water by shaking for 1 h using an Intelli-Mixer (60 r.p.m.). The extracts were centrifuged for 12 min (4°C, 4500 r.p.m.) and stored at −20°C. Details on method development and performance are included in §2.8.

#### 2.6.3. Larvae

Extractions of the pooled larval samples were performed with 10 mg of the third instar and 15 mg of the fourth and fifth instar samples. The extraction protocols for gelsemine, atropine, amygdaline, senkirkine, methyllycaconitine and triptolide were optimized from protocols developed for adult honey bees [57]. Initial tests with the adult bee protocols did not provide adequate results for larval samples and were modified as described in the following text. Details on method adaptation to larval samples and performance evaluation are included in §2.8.

Amygdalin and atropine were extracted with 1 ml of 7:3 methanol:water + 0.2% acetic acid, while gelsemine, senkirkine and methyllycaconitine were extracted with 1 ml of 1:1 methanol:water. The shaking extractions were performed using an Intelli-Mixer (60 r.p.m.) for 1 h. The extracts were centrifuged for 12 min (4°C, 4500 r.p.m.) and stored at −20°C. Flavonoids and phenolic acids were extracted by 1 h shaking extraction with 1 ml 7:3 methanol:water as described in §2.6.2 for adult honey bee samples.

### 2.7. HPLC-MS/MS analyses and quantification

Prior to HPLC-MS/MS analyses, all larvae, adult bee and pollen extracts were diluted with water and filtered using a syringe filter (Kinesis KX PTFE syringe filter 13 mm, 0.22 μm, Mikrolab, Aarhus, Denmark).

The 12 phenolic acids and the six artificially added PSMs (electronic supplementary material, table S2) were quantified using an Agilent 1260 Infinity HPLC (Agilent Technologies, Glostrup, Denmark) coupled with a Sciex (Copenhagen, Denmark) 4500 QTRAP mass spectrometer equipped with electrospray ionization and operated in multiple reaction monitoring (MRM) mode. The instrument was controlled by Analyst software version 1.6.3. Nitrogen was used as source and collision gas. The compound-dependent mass spectrometer parameters (declustering potential (DP), entry potential (EP), cell entry potential (CEP), collision energy (CE) and cell exit potential (CXP)) that were optimized by infusing the pure compound directly into the mass spectrometer source are listed in electronic supplementary material, table S3, together with the two MRM transitions monitored for each compound. The MRM giving the highest response was used for quantification, and the other was used as a qualifier to ensure correct identification. The phenolic acids were analysed in negative mode with chromatographic separation on a Phenomenex Kinetex Polar C18 column (100 × 2.1 mm, 2.6 μm, 100 Å; Phenomenex, Værløse, Denmark) with a flow rate of 0.4 ml min^–1^ and mobile phases A: 92% water + 4% methanol + 4% 2-propanol + 20 mM acetic acid and B: 92% acetonitrile + 4% methanol + 4% 2-propanol + 20 mM acetic acid. The gradient was as follows: 0–2 min: 100% A; 2–3.6 min: 100–54.4% A; 3.6–3.8 min: 54.4–16.3% A; 3.8–4.5 min: 16.3% A; 4.5–4.7 min: 16.3–100% A; 4.7–8 min: 100% A. The column oven was set to 30°C, and the injection volume was 10 µl. The mass spectrometer source parameters were curtain gas (CUR), 35 psi; collision gas (CAD), medium; temperature (TEM), 600°C; ion source gas 1 (GS1), 90 psi; ion source gas 2 (GS2), 90 psi; and ionspray voltage (IS), −4300 V. Standard curves were recorded in the concentration interval 0.048–800 ng ml^–1^. Gelsemine, atropine, senkirkine, methyllycaconitine and triptolide were analysed in positive mode and amygdalin in negative mode using chromatographic conditions and mass spectrometer source parameters as described in Vidkjær *et al*. [57]. Standard curves were recorded in the concentration interval 0.0015–50 ng ml^–1^ for amygdalin, gelsemine, atropine, senkirkine and methyllycaconitine and 0.006–2.5 ng ml^–1^ for triptolide. Quantifications were performed in Analyst software version 1.6.3.

The 29 flavonoids (electronic supplementary material, table S2) were quantified using an Agilent 1260 Infinity HPLC (Agilent Technologies, Glostrup, Denmark) coupled with a Sciex (Copenhagen, Denmark) 3200 QTRAP mass spectrometer equipped with electrospray ionization and operated in MRM mode. The instrument was controlled by Analyst software version 1.6.3. The chromatographic conditions and mass spectrometer source parameters were as previously published [62]. The optimized compound-dependent mass spectrometer parameters (DP, EP, CE, CXP) are listed in the electronic supplementary material, table S3, together with the two MRM transitions monitored and the ionization mode applied for each flavonoid. Standard curves were recorded in the concentration interval 0.39–800 ng ml^–1^, and quantifications were performed in Analyst software version 1.6.3.

### 2.8. Analytical method development and validation

The analytical protocols for adult honey bees and their larvae were developed by spiking known concentrations of pure PSMs into dried pulverized honey bee and larval samples to test different extraction protocols. The bees and larvae used for method development did not originate from the experimental hives and were confirmed to be free of the targeted PSMs by HPLC-MS/MS. All six artificially added PSMs and a subset of flavonoids and phenolic acids were used to develop and evaluate the performance of the analytical protocols.

To validate the optimized analytical protocols, accuracy and precision were tested by spiking two separate concentrations of the pure PSMs into dried pulverized adult honey bees (*n* = 5) and larvae (*n* = 6) free from the targeted PSMs. Recovery percentages, representing the percentage of the theoretical concentration measured in the spiked samples, were calculated to evaluate method accuracy, while precision was assessed by the relative standard deviation in per cent (RSD%) between replicate samples. The limits of detection (LOD) and quantification (LOQ) were set as the standard concentrations for which the signal-to-noise ratios (*S*/*N*) were equal to or above 3 and 10, respectively. Method validation details and data are provided in the electronic supplementary material (tables S4 and S5). Briefly, the recovery percentages for flavonoids and phenolic acids ranged from 51 to 98% for adult bees and 59–116% for larvae. RSD% were 4–16% and 3–17% for adult bees and larvae, respectively. The methods for the six PSMs artificially added to the diet were previously validated for adult honey bees, as detailed in Vidkjær *et al*. [57]. The methods adopted for analysing these compounds in larval samples were validated as part of this experiment, with recovery percentages ranging from 63 to 134% and RSD% from 2 to 12%. The recovery percentages were not used to correct the concentrations measured in adult bees and larvae.

### 2.9. Microscopic analyses of pollen in larvae guts

Fourth and fifth instar larvae were homogenized individually (*n* = 5) in an Eppendorf^®^ tube with a fitting micropestle^®^ and 100 µl of Milli-Q water. Due to their smaller size, the third instar larvae were homogenized in a pool of ten larvae with 200 µl of Milli-Q water. Microscopic examinations of the homogenates were done with phase contrast at 100× to determine the presence or absence of pollen grains (electronic supplementary material, figures S2–S4). Identification of the pollen species as clover was done at 300× magnification (electronic supplementary material, figure S5).

### 2.10. Data analyses and statistics

Data analyses and statistics were performed using R version 4.0.4 and the R Base Package. Changes in PSM diversity across diet, adult bees, and larvae were assessed by comparing the number of PSMs detected in these samples by one-way analyses of variance (ANOVA; *α* = 0.05) followed by Tukey’s test for *post hoc* analyses (figure 2; see electronic supplementary material, table S6, for statistical output). FDR-corrected *p*-values were used to adjust for multiple comparisons. Principal component analyses (PCA; data centred and scaled) were performed to explore changes in PSM composition between the diet and the adult bees/larvae as well as between adult bee and larval stages. Prior to PCA, concentration data were converted to percentages of the total PSM concentrations in each sample (electronic supplementary material, table S7). This approach allowed for the comparison of the compositional distribution of individual PSMs across pollen, adult bees and larvae rather than comparing raw concentrations, which varied significantly between the sample groups. The linear relationship between dietary PSM concentrations and the concentrations measured in the larvae and adult bees was explored for each of the three PSM groups (flavonoids, phenolic acids, added PSMs) by plotting the diet concentrations versus the concentrations in adult bees and larvae (third, fourth and fifth instar larvae plotted separately) (electronic supplementary material, figure S6). Linear regression was used to fit the data points, and linearity was assessed using the *r* ^2^ value. To test how the concentrations of individual PSMs change with larval age, the fold-change (FC) values between instars (third versus fourth, fourth versus fifth and third versus fifth) were calculated for each PSM (electronic supplementary material, table S8). Only larvae from the same miniature hive were compared, as the specific FC values differed between the hives. For each hive, one-way ANOVAs followed by Tukey’s test for *post hoc* analyses (*α* = 0.05) were used to determine if the concentration changes represented by the FC values were significant (electronic supplementary material, table S9). Statistical outputs are included in electronic supplementary material, table S10. FDR-corrected *p*-values were used to adjust for multiple comparisons.

## 3. Results and discussion

### 3.1. Flavonoids and phenolic acids in white clover pollen

**Figure 1.**
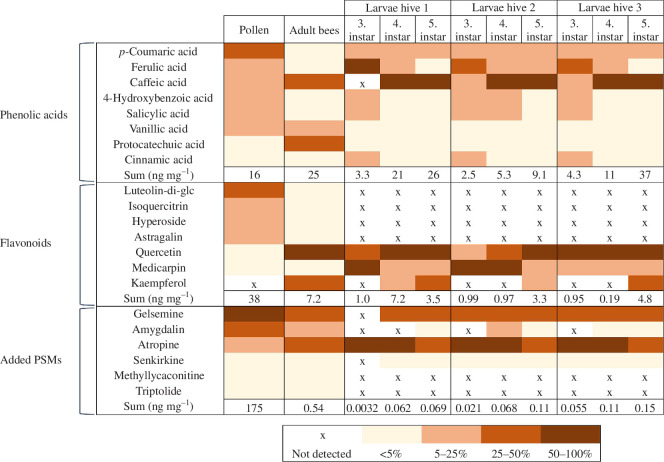
Plant secondary metabolites (PSMs) in pollen diet, adult honey bees and larvae. Heatmap showing the distribution of PSMs in pollen diet, adult bees and larvae from the three miniature hives (H1, H2, H3). The colours indicate the per cent distribution of each compound, calculated as the mean percentage of the total concentration of PSMs within the three compound groups: flavonoids, phenolic acids and PSMs artificially added to the clover pollen diet. The numbers represent the summed mean concentrations of each compound group within the samples. Only the most abundant flavonoids and phenolic acids, with concentrations ≥5% in at least one sample, are included. Data for the remaining flavonoids and phenolic acids are provided in electronic supplementary material, tables S11 and S12, along with individual concentrations of all PSMs analysed by HPLC-MS/MS.

Approximately 90% of the pollen flavonoids were *O*-glycosides of luteolin (luteolin−3′,7-di-*O*-glucoside, hereafter luteolin-di-glc), quercetin (isoquercetrin and hyperoside) and kaempferol (astragalin) (figure 1). Rutin, quercetin, naringenin, nicotiflorin, quercetin-xyl-gal (quercetin−3-*O*-{β-d-xylosyl-(1→2)-β-d-galactoside}), kaempferol-rha-glc (kaempferol−7-*O*-{α-l-rhamnosyl-d-glucoside}), medicarpin, and genistin had low concentrations from 0.06 to 2.7 ng mg^–1^ pollen (0.16–7.2% of the pollen flavonoids). Biochanin A, formononetin, luteolin, kaempferol-rha-xyl-gal (kaempferol−3-*O*-{β-d-rhamnosyl-(1→6)-[β-d-xylosyl-(1→2)]-β-d-galactoside}), quercetin-rha-xyl-gal (quercetin−3-*O*-{β-d-rhamnosyl-(1→6)-[β-d-xylosyl-(1→2)]-β-d-galactoside}) and isovitexin were present at trace levels (figure 1; electronic supplementary material, table S11). Quercitrin, daidzein, saponarin, sissotrin, luteolin-glc (luteolin−4′-*O*-glucoside), puerarin, daidzin, coumestrol, genistein and kaempferol, which have previously been identified in flowers, leaves or stems of white clover plants [42], were not detected in the pollen. The pollen phenolic acids were primarily composed of three cinnamic acid derivatives, *p*-coumaric, ferulic and caffeic acids, and two benzoic acid derivatives, 4-hydroxybenzoic and salicylic acids (figure 1) (collectively ~88%). Salicylic, vanillic, gallic, protocatechuic, cinnamic and sinapic acids had low concentrations from 0.1 to 0.99 ng mg^–1^ pollen (0.61–6.1% of the phenolic acids) (figure 1; electronic supplementary material, table S11) while chlorogenic and syringic acids were detected at trace levels (electronic supplementary material, table S11).

### 3.2. Plant secondary metabolites from pollen recovered in adult honey bees

**Figure 2.**
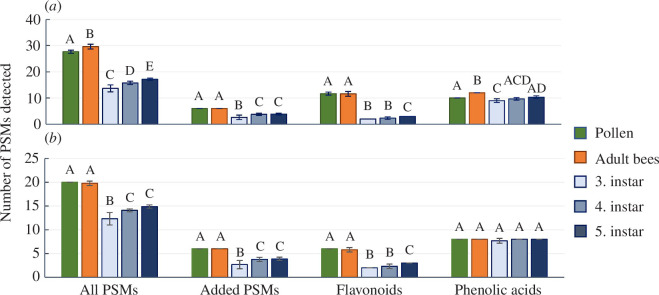
Plant secondary metabolite (PSM) diversity differs between pollen diet, adult honey bees and larvae. Diversity is measured as the number of detected PSMs: (*a*) includes all PSMs and (*b*) compares the most abundant PSMs with concentrations ≥5% in any of the sample groups. Bars represent mean values, with error bars indicating standard deviation. Compound diversity was evaluated separately for each of the four PSM groups—all PSMs, added PSMs, flavonoids and phenolic acids—by one-way ANOVAs (electronic supplementary material, table S6). Letters above the bars denote significance and for each PSM group, bars having letters in common are not significantly different.

**Figure 3.**
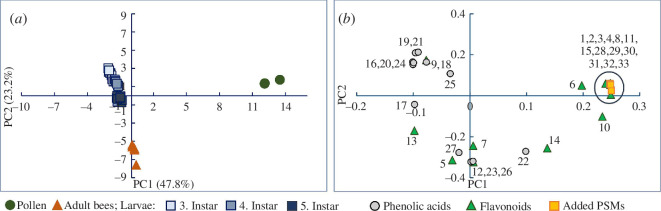
Comparison of plant secondary metabolite (PSM) profiles in pollen diet, adult honey bees and larvae. (*a*) The PCA scores plot shows distinct PSM profiles of adult bees and larvae that differ from their pollen diet (adult bees, *n* = 5 pools; larvae, *n* = 3 pools per hive and instar; pollen, *n* = 3). The PCA loadings plot (*b*) shows that the pollen diet is characterized by flavonoid glycosides and the six artificially added PSMs, while adult bees and larvae primarily contain phenolic acids and a few flavonoid aglycones. The numbers correspond to individual PSMs: 1, astragalin; 2, genistin; 3, hyperoside; 4, isoquercitin; 5, kaempferol; 6, kaempferol-rha-glc; 7, luteolin; 8, luteolin-di-glc; 9, medicarpin; 10, naringenin; 11, nicotiflorin; 12, pratensein; 13, quercetin; 14, quercetin-xyl-gal; 15, rutin; 16, 4-hydroxybenzoic acid; 17, caffeic acid; 18, chlorogenic acid; 19, cinnamic acid; 20, coumaric acid; 21, ferulic acid; 22, gallic acid; 23, protocatechuic acid; 24, salicylic acid; 25, sinapic acid; 26, syringic acid; 27, vanillic acid; 28, amygdalin; 29, atropine; 30, gelsemine; 31, methyllycaconitine; 32, senkirkine; 33, triptolide.

The diversity of PSMs in the young adult honey bees was significantly higher (figure 2*a*) than the pollen diet, but the difference was driven by two phenolic acids with low bee abundance (≤5%) that were only detected at trace levels in the pollen (chlorogenic acid and syringic acid). When PSMs with ≤5% abundance were removed, the difference in PSM diversity became non-significant (figure 2*b*). The PCA showed that the PSM compositions of adult bees differed from the pollen diet (figure 3). Flavonoid glycosides and the six artificially added PSMs were dominant in the pollen, while phenolic acids and flavonoid aglycones had the highest concentrations in the bees (figure 3). Approximately 90% of the bee flavonoids were the aglycones, quercetin and kaempferol. Quercetin was only a minor component of the pollen diet (2%), whereas kaempferol was not detected (figure 1 and electronic supplementary material, table S11). We conclude that the bee flavonoid aglycones originate from dietary *O*-glycosides of quercetin and kaempferol that were de-glycosylated in the bees following ingestion. The main pollen flavonoid, luteolin-di-glc, was not recovered in the bees, and the corresponding aglycone, luteolin, was only a minor component (~1%). The remaining pollen flavonoids were either absent or had low concentrations in bees (0.2–3.5%) (figure 1 and electronic supplementary material, table S11).

All pollen phenolic acids were recovered in adult bees, but the compound per cent distribution differed from the diet (figures 1 and 3). The bees mainly contained protocatechuic, vanillic and caffeic acids (~87% of the total phenolic acid concentration) (figure 1). Of these, only caffeic acid had high pollen abundance (16%), whereas protocatechuic and vanillic acids were minor components (≤6%). The remaining nine pollen phenolic acids had low bee concentrations (0.05–3.9%), including the pollen phenolic acid with the highest concentration, *p*-coumaric acid (figure 1; electronic supplementary material, table S11). All six PSMs artificially added to the diet were recovered in the adult bees (figure 1 and electronic supplementary material, table S11). Gelsemine, amygdalin and atropine that were added to the pollen diet in higher concentrations were more abundant in the bees than the PSMs added in lower concentrations (senkirkine, methyllycaconitine and triptolide) (figure 1). However, when examining the relationship between diet and bee concentrations, the trend was not linear. The *r* ^2^ value was 0.76 for the added PSMs (electronic supplementary material, figure S6) but atropine was for instance detected in the bees in higher concentrations than amygdalin even though the diet atropine concentration was 2.5 times lower than amygdalin. Furthermore, the content of methyllycaconitine in bees was approximately six times higher than senkirkine and triptolide, although the diet methyllycaconitine concentration was the same as triptolide and three times lower than senkirkine (figure 1 and electronic supplementary material, table S11). For the clover flavonoids and phenolic acids, there were no indications of a linear relationship between diet and bee concentrations, and the *r* ^2^ values were 0.14 and 0.10, respectively (electronic supplementary material, figure S6). Variations in uptake and/or differences in post-ingestion metabolization between individual PSMs likely explain why high dietary concentrations of specific PSMs do not necessarily result in high bee concentrations. These results are in agreement with our previous findings [57].

**Figure 4.**
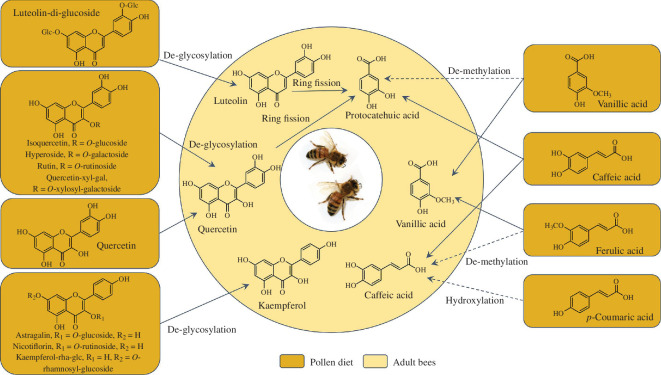
Proposed metabolization pathways for the most abundant clover pollen phenolic acids and flavonoids in adult honey bees. Unbroken arrows represent well-established biotransformation pathways, while dashed arrows indicate hypothesized pathways. The proposed pathways are based on mammalian plant secondary metabolite (PSM) metabolism [69–73], as detailed knowledge of PSM biotransformations in bees and other insects is currently lacking.

Flavonoids and phenolic acids have been recovered in several insect species. Flavonoids are predominantly recovered as glycosides [63–67], which may originate from the diet or be formed post-ingestion by glycosidation. The conjugation of dietary flavonoid aglycones can function as an insect detoxification mechanism [68]. Contrary to these observations, our data show that honey bees extensively de-glycosylate flavonoids post-ingestion (figure 4). Honey bees secrete glycoside hydroxylases from their hypopharynx glands into their mouth. These enzymes are transmitted to the midgut, where they may catalyse de-glycosylation of flavonoids [74,75]. Gut microorganisms may further contribute to PSM metabolization and work in conjunction with the honey bees’ own enzyme systems [29,30]. The grasshopper, *Tropidacris collaris*, also de-glycosylates dietary flavonoid conjugates and this was suggested to serve as an important carbohydrate source [76]. It is not known if flavonoid de-glycosylation has nutritional value for honey bees, and nectar serves as their primary carbohydrate source. However, de-glycosylation of dietary flavonoids may provide additional carbohydrates not found in nectar, which predominantly consists of sucrose, fructose and glucose [77]. Some flavonoids are known to positively impact honey bee disease resistance and pesticide tolerances and understanding their metabolic fate can facilitate mechanistic insight. We for example recently linked several dietary flavonoid glycosides to enhanced pesticide detoxification in honey bees [26]. Based on the results of the present experiment, we hypothesize that this increase in pesticide metabolism was caused by the respective aglycones formed post-ingestion that increase the expression of P450 genes similarly to quercetin [23].

The main pollen flavonoid, luteolin-di-glc, and its aglycone, luteolin, were only detected in low concentrations in the bees (figure 1). We hypothesize that the low luteolin abundance, compared with quercetin and kaempferol, arises from differences in how honey bees metabolize the structurally different flavonol (quercetin and kaempferol) and flavone (luteolin) aglycones (figure 4). Flavonones may be converted to downstream metabolites faster than the two flavonols, leading to lower luteolin abundance. This is in agreement with our previous documentation of substantial variation in the degradation ratios of structurally different PSMs in honey bees [57]. The metabolic fate of the flavonoid ring system remains virtually unexplored in insects. Elucidation of full pathways would require a different analytical approach from that utilized in this experiment. However, mammalian degradation of the flavonoid aglycone ring system produces various phenolic acids, including protocatechuic, caffeic and vanillic acids [69–72] that were targeted by our analyses. Protocatechuic acid, a well-established mammalian metabolite of luteolin [69–71], was the most abundant phenolic acid in the bees (47%) but only a minor component of the diet (~1%). We hypothesize that the high concentrations we documented in the adult honey bees originate from ring-system cleavage of dietary flavonoids in the bees, mainly from luteolin-di-glc (figure 4). The other luteolin metabolites, caffeic and vanillic acids, were the second and third most abundant phenolic acids in the bees, but these were ingested in higher concentrations. Metabolization of dietary cinnamic acids may also have contributed to the formation of protocatechuic and vanillic acids in the bees as these compounds are also known metabolites of caffeic and ferulic acids, respectively (figure 4) [72,73]. Honey bees possess flavonoid-metabolizing cytochrome P450 enzymes [78] but metabolites originating from flavonoid ring-system cleavage were recently identified in the honey bee gut [29], suggesting that gut microorganisms may work in synergy with the bees’ own enzyme system. Based on our data, it is not possible to determine where in the bees these metabolites were formed.

Metabolization pathways for the PSMs artificially added to the clover pollen diet have not yet been clarified in honey bees or any other insect except for amygdalin metabolism, which was recently determined in honey bees [30]. We previously tested the bioavailability and gut uptake of these compounds in adult honey bees [57]. In this experiment, we included these PSMs to test if they are passed on to the brood and to compare bee concentrations of the added PSMs that are produced by a few plant species to concentrations of flavonoids and phenolic acids, which are omnipresent in pollen and nectar. Interestingly, all artificially added PSMs had lower adult bee concentrations than flavonoids and phenolic acids, even though they were consumed in significantly higher concentrations.

### 3.3. Plant secondary metabolites from pollen recovered in honey bee larvae

Larval PSM diversity was significantly reduced compared with the pollen diet, except for the phenolic acids (figure 2). All phenolic acids with a pollen abundance ≥5% were recovered in the larvae (figures 1 and 2*b*). The PCA showed that larvae have distinct PSM profiles primarily composed of a few flavonoid aglycones and a mix of phenolic acids (figure 3), differing from pollen PSM profiles (figure 3). None of the pollen flavonoid glycosides were detected in the larvae, and medicarpin and quercetin were the only flavonoids consistently present across all three instars (figures 1 and 2; electronic supplementary material, table S12). Kaempferol was also detected in the fifth instar larvae and in the fourth instar larvae from hive 1 (figure 1). It is unclear if larvae can perform de-glycosylations. In this experiment, the jelly was not analysed but work by others has identified flavonoid glycosides in jelly [37], pointing to aglycones being formed in the larvae and not prior to the transmission into nurse bee jelly. The most abundant phenolic acids in the third instar were cinnamic, salicylic, 4-hydroxybenzoic, ferulic, *p*-coumaric and caffeic acids (figure 1 and electronic supplementary material, table S12). In the fourth and fifth instar, the abundance of caffeic and *p*-coumaric acids increased, making these the main phenolic acids along with ferulic acid (figure 1). Of the six compounds artificially added to the pollen diet, only gelsemine, atropine, amygdalin and senkirkine were recovered in the larvae (figure 1). Methyllycaconitine and triptolide, which had the lowest concentration in the diet, were not detected. This suggests that compound concentrations in the nurse bees’ diet influence PSM transmission to the brood, but neither the artificially added PSMs nor the clover pollen phenolic acids and flavonoids had a linear relationship with larval PSM concentrations. For all PSM groups, the *r* ^2^ value was between 0.1 and 0.3 regardless of larva instar (electronic supplementary material, figure S6). PSM concentrations differed between larvae collected from the three miniature hives (figure 1). We attribute this to variations in nurse bee food intake between the hives (total intake day 1–5: H1, 24 mg per bee; H2, 21 mg per bee; H3, 25 mg per bee), unequal numbers of larvae, and differences in jelly quantities provisioned by the nurses. Despite differences in absolute concentrations, the per cent distribution across each PSM group was similar across the hives (figures 1 and 2).

In third instar larvae, pollen grains were absent, except for one larva, which contained two pollen grains. Fourth instar larvae contained 1–3 pollen grains while the fifth instar had 10–20 clover pollen grains per larvae. Representative images from the analyses are included in electronic supplementary material, figures S2–S5. Since larvae cannot produce PSMs and the third instar larvae were pollen-free, we conclude that the PSMs detected in the early instar originate from jelly provisioned by nurses. The diverse PSM profile further documents that various pollen PSMs are indeed passed to the brood via the jelly. However, the significantly reduced larval PSM diversity (figure 2) suggests that some dietary components are not transmitted. PSMs in later instars may originate from either jelly or pollen as small amounts of pollen grains were identified in the guts of fourth and fifth instar larvae. Given the diversity of compounds detected in the pollen-free third instar larvae and the few pollen grains found in the older larvae, we conclude that jelly is the main PSM transmission pathway.

Transmission of dietary components into nurse bee jelly has only been studied for a few xenobiotic compounds. The concentrations of the pyrrolizidine alkaloid echimidine in jelly was reduced by three orders of magnitude compared with the pollen ingested by the nurses [36]. Similarly, several pesticides show reduced concentrations in jelly, while others seem to be absent despite being consumed by the nurses [79]. These observations suggest that xenobiotic transmission into jelly is highly compound-dependent and that jelly xenobiotic concentrations are significantly lower than in the nurse bees’ diet. This has led to the hypothesis that nurse bees feeding larvae jelly instead of pollen and nectar serves as a protective mechanism, reducing brood exposure to potentially toxic compounds [36]. Toxicity is rarely assessed in both adult bees and brood but larvae appear to be more sensitive to potentially toxic PSMs [36], and the activity of cytochrome P450s, mediating xenobiotic detoxification, is low in early instar larvae [80]. However, differences in toxic effects between adult bees and larvae may vary significantly depending on the compound. For example, larvae are less tolerant than adult bees to the pyrrolizidine alkaloid echimidine [36] but not to nicotine [81]. In this study, we found significantly reduced larval diversity of flavonoids and the PSMs artificially added to the diet. These results support the notion of reduced transmission of these PSMs into the jelly, thereby minimizing larval exposure to some dietary components. In contrast, all dietary phenolic acids were recovered in the larvae, suggesting that these PSMs are generally passed. Flavonoids and phenolic acids were also more abundant in the larvae than the PSMs artificially added to the diet, even though the latter had the highest diet concentration (figure 1). These results do not support a general protective effect of nurses feeding the larvae jelly that reduces larval concentrations across all PSMs but rather point to a highly compound-dependent PSM exposure of the brood. We did not measure the jelly PSM composition, so we cannot rule out that the discrepancies in PSM diversity between larvae and pollen diet might also be driven by differences in larval metabolization of individual PSMs or a combination of both. The ability of honey bee larvae to metabolize PSMs is poorly understood, but nicotine degradation was recently documented in late instar larvae [81], while early instars have not been tested.

**Figure 5.**
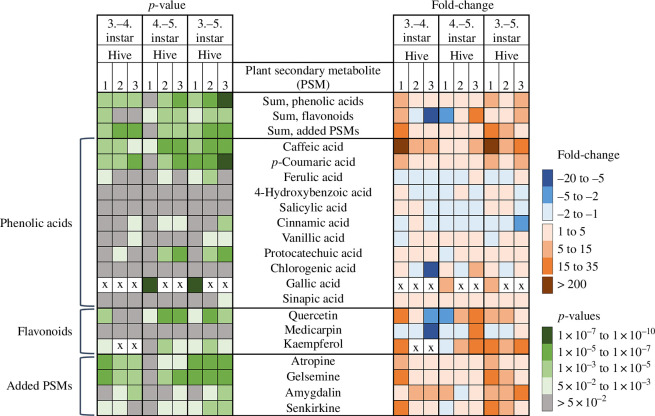
Changes in plant secondary metabolite (PSM) concentrations with larval age. The concentrations of several PSMs increase significantly with larval age from the third to the fifth instar. The right side of the heatmap shows fold-change (FC) values for each miniature hive (H1, H2, H3), indicating changes in larval concentrations between instars. The left side displays the *p*-values from one-way ANOVAs used to determine if the concentration changes within each hive were significant. PSMs that were not detected are labelled with an ‘x’. FC and *p*-values are provided in electronic supplementary material, tables S8 and S9, while the statistical outputs are in electronic supplementary material, table S10.

Interestingly, our results showed that the concentration of several PSMs significantly increased from the third to the fifth larval instar. Notable increases in FC were observed for quercetin (FC 5–9), caffeic acid (FC 12–400), *p*-coumaric acid (FC 4–12), atropine (FC 2–14), gelsemine (FC 4–28), amygdalin (FC 10–17) and senkirkine (FC 2–16) (numbers in parentheses indicate FC ranges across H1, H2, H3; see figure 5 and electronic supplementary material, table S8). Although FC values varied among individual hives, the trends were consistent, particularly between the third and the fifth instars (figure 5). These results suggest that some PSMs accumulate in larvae as they develop. Few PSMs showed negative FC, indicating a decrease in concentration, and these decreases were generally non-significant (figure 5). During larval development, PSM depletion can only happen via metabolic degradation, as the midgut is not connected to the hindgut until the pre-pupal stage, causing undigested material to accumulate in the midgut [82]. We hypothesize that some PSMs increase in concentration because the larvae either metabolize these compounds very slowly or not at all, leading to accumulation across instars. Continuous PSM exposure could eventually lead to larval concentrations exceeding thresholds for acute toxicity or sub-lethal negative impacts on larval health. The PSMs artificially added to the pollen diet can be toxic to adult bees [5,54] and all increased with larval age (atropine, gelsemine, amygdalin and senkirkine). Toxic effects are highly concentration-dependent and exposure to sub-lethal levels may even be health-promoting. For example, amygdalin has an adult honey bee LD_50_ of 0.003% [5] but can reduce virus titres [83] and trypanosomatid parasite *Lotmaria passim* infections [83] at lower concentrations. Thus, the accumulation of dietary PSMs in larvae may alter bioactivities and potentially lead to detrimental toxic effects. When honey bees have access to a diverse range of forage plants, the consumption of individual potentially toxic PSMs is likely diminished as the bees mix pollen and nectar from several plant species with different PSM compositions. However, if they continuously forage on plants containing toxic substances, e.g. when plant species diversity is low, this increases exposure to potentially problematic dietary components. This scenario may pose a higher risk to the brood if some PSMs accumulate. Negative impacts will depend on larval toxicity thresholds, for which virtually no data exist. Our results emphasize the need to determine larval PSM tolerances, especially considering changing floral resources due to season, weather conditions, changes in land use, climatic shifts or transhumance of colonies by beekeepers, all of which could significantly alter PSM exposure.

Flavonoids and phenolic acids were the most abundant PSMs in the larvae. Unlike the artificially added PSMs, these compounds appear to have low toxic potential and are associated with health-promoting activities in adult honey bees [10,15,23]. Dominant larval PSMs like caffeic acid, *p*-coumaric acid and quercetin can promote metabolism [25,84] and increase the LD_50_ values of some pesticides in adult honey bees [23]. Although the influence of these compounds on pesticide detoxification in larvae is not yet determined, Mao *et al*. [80] found that quercetin feeding upregulated the expression of the same P450 genes in both larvae and adult honey bees, suggesting similar impacts on pesticide detoxification. Numerous studies have reported pesticide contamination of honey bee bread and wax combs [85–88], documenting pesticide exposure of the brood. Honey bees reared in pesticide-contaminated combs experience delayed larval development, delayed emergence and reduced longevity [89]. Honey bee nurses feeding on pollen that supplies the larvae with biologically adequate levels of *p*-coumaric acid, quercetin, and potentially other structurally related compounds may mitigate these effects by enhancing brood pesticide tolerance and detoxification. Flavonoids and phenolic acids also impact other aspects of honey bee health [18,90,91], such as upregulating innate immunity genes and genes encoding for the production of antimicrobial peptides in both adults and larvae [14,15]. The concentration dependence of these activities is not yet determined, and the increasing concentrations of caffeic acid, *p*-coumaric acid and quercetin with larval age documented by this study may alter their effects from health promoting to harmful. For example, the accumulation of quercetin in adult honey bees exposed to a triazole fungicide that inhibits quercetin metabolism impaired the bees’ ATP energy production [80].

### 3.4. Comparison of plant secondary metabolite profiles between adult honey bees and larvae

**Figure 6.**
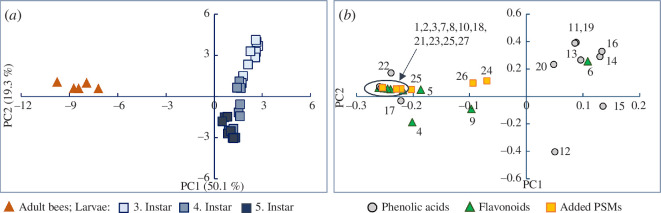
Comparison of plant secondary metabolite (PSM) profiles of adult honey bees and larvae. The PCA scores plot (*a*) shows distinct PSM profiles of adult bees and larvae (bees, *n* = 5 pools; larvae, *n* = 3 pools per hive and instar). The loadings plot (*b*) shows that larval stages are predominantly characterized by phenolic acids, whereas adult bees exhibit higher concentrations of flavonoids and the PSMs that were artificially added to the diet. Numbers correspond to individual PSMs: 1, astragalin; 2, hyperoside; 3, isoquercitin; 4, kaempferol; 5, luteolin; 6, medicarpin; 7, naringenin; 8, pratensein; 9, quercetin; 10, quercetin-xyl-gal; 11, 4-hydroxybenzoic acid; 12, caffeic acid; 13, chlorogenic acid; 14, cinnamic acid; 15, coumaric acid; 16, ferulic acid; 17, gallic acid; 18, protocatechuic acid; 19, salicylic acid; 20, sinapic acid; 21, syringic acid; 22, vanillic acid; 23, amygdalin; 24, atropine; 25, gelsemine; 25, methyllycaconitine; 26, senkirkine; 27, triptolide.

Larval PSM diversity was significantly reduced compared with adult bees for all compound groups except phenolic acids, with an abundance above 5% (figure 2). These differences were mainly driven by flavonoids and the two artificially added compounds, triptolide and methyllycaconitine, which were absent in larvae and had low concentrations in adult bees. The PCA showed a clear separation between larval and adult bee PSM profiles. Phenolic acids were dominant in larvae, while adult bees contained higher levels of flavonoids and the PSMs artificially added to the diet (figure 6). The differences between adult bees and larvae are likely due to variations in the transmission of dietary PSMs to the brood and differences in PSM metabolism between developmental stages. We consistently detected lower concentrations of some of the flavonoids and all artificially added PSMs in larvae compared with adult bees (figures 1 and 6), supporting the proposed protective effect of nurses feeding larvae jelly to reduce larval PSM exposure (discussed in §3.3). However, several phenolic acids had the highest concentration in larvae, and some larval samples also exceeded adult bee concentrations of medicarpin and quercetin (electronic supplementary material, tables S11 and S12). These observations suggest that PSM transmission via jelly is highly compound-dependent rather than indicating a general protective effect of honey bee nursing.

The flavonoid ring-system degradation products, vanillic and protocatechuic acids, detected in high concentrations in adult bees were only minor components in larvae (figure 1). This suggests different larval flavonoid metabolization pathways or an inability of larvae to metabolize the flavonoid ring system. This is consistent with the observed increase in flavonoid aglycones from the third to the fifth larval instar (figure 5). Differences in xenobiotic metabolism between adult bees and larvae remain largely unexplored, especially for PSM xenobiotics. However, metabolization of the agrochemical formulant *N*-methyl-2-pyrrolidone was faster in adult bees than in larvae, which also formed different metabolites from adult bees [92]. Pathway and rate differences between larvae and adult bees likely reflect changes in enzyme activity documented across developmental stages [92]. Each developmental stage of honey bees also has a distinct gut microbial composition [93,94], with larval guts possessing few or no bacteria [95]. Since gut microbiota plays a role in degrading flavonoids [29] and other dietary PSMs [30], the near-absence of gut microbiota in larval stages may contribute to the substantial decrease in flavonoid ring-system metabolites compared with adult bees documented by our study.

## 4. Conclusion

This experiment presents the first documentation of pollen diet PSMs from multiple compound classes in honey bee larvae and the first comparison of diet PSMs across honey bee developmental stages. Our results reveal distinct PSM profiles of adult honey bees and three larval stages, differing from their pollen diet composition. These differences are attributed to post-ingestion PSM metabolism and variations in the transmission of individual PSMs to the brood via nurse bee jelly. Both bees and larvae had the highest concentrations of phenolic acids and flavonoid aglycones, which are naturally present in the clover pollen diet and omnipresent in pollen and nectar. In contrast, the alkaloids, cyanogenic glycoside and diterpene produced by fewer plant species were less abundant in both adult bees and larvae despite being consumed in higher concentrations.

Our findings propose several new hypotheses about PSM fate in adult honey bees and their larvae, with potential implications for honey bee health. Notably, our results point to differences in flavonoid metabolism between adult bees and larvae and that PSM transmission to the brood via nurse bee jelly is highly compound-dependent. The increasing concentrations of several PSMs with larval age suggest a reduced ability of honey bee larvae to degrade certain dietary PSMs, leading to accumulation and potential negative effects on brood health. Future experiments should focus on these hypotheses to improve our understanding of PSM metabolism and the impacts of dietary PSMs on honey bee health.

## Data Availability

The HPLC-MS/MS data tables are included as electronic supplementary material [96]. The raw HPLC-MS/MS data files are available via Dryad [97].
